# Preoperative Pain Catastrophizing and Neuropathic Pain Do Not Predict Length of Stay and Early Post-Operative Complications following Total Joint Arthroplasty

**DOI:** 10.3390/jpm13020216

**Published:** 2023-01-26

**Authors:** Shai S. Shemesh, James Douglas Dieterich, Darwin Chen, Roni Sharon, Michael J. Bronson, Tal Frenkel Rutenberg, Calin S. Moucha

**Affiliations:** 1 Department of Orthopedic Surgery, Rabin Medical Center, Sackler School of Medicine, Tel Aviv University, Tel Aviv 6997801, Israel; 2 Department of Orthopedic Surgery, Icahn School of Medicine at Mount Sinai, New York, NY 10029, USA; 3 Department of Neurology, Sheba Medical Center, Sackler School of Medicine, Tel Aviv University, Tel Aviv 6997801, Israel

**Keywords:** arthroplasty, hip, knee, catastrophizing, pain, PainDETECT, neuropathic

## Abstract

Background: Both pain catastrophizing and neuropathic pain have been suggested as prospective risk factors for poor postoperative pain outcomes in total joint arthroplasty (TJA). Objective: We hypothesized that pain catastrophizers, as well as patients with pain characterized as neuropathic, would exhibit higher pain scores, higher early complication rates and longer lengths of stay following primary TJA. Methods: A prospective, observational study in a single academic institution included 100 patients with end-stage hip or knee osteoarthritis scheduled for TJA. In pre-surgery, measures of health status, socio-demographics, opioid use, neuropathic pain (PainDETECT), pain catastrophizing (PCS), pain at rest and pain during activity (WOMAC pain items) were collected. The primary outcome measure was the length of stay (LOS) and secondary measures were the discharge destinations, early postoperative complications, readmissions, visual analog scale (VAS) levels and distances walked during the hospital stay. Results: The prevalence of pain catastrophizing (PCS ≥ 30) and neuropathic pain (PainDETECT ≥ 19) was 45% and 20.4%, respectively. Preoperative PCS correlated positively with PainDETECT (rs = 0.501, *p* = 0.001). The WOMAC positively correlated more strongly with PCS (rs = 0.512 *p* = 0.01) than with PainDETECT (rs = 0.329 *p* = 0.038). Neither PCS nor PainDETECT correlated with the LOS. Using multivariate regression analysis, a history of chronic pain medication use was found to predict early postoperative complications (OR 38.1, *p* = 0.47, CI 1.047–1386.1). There were no differences in the remaining secondary outcomes. Conclusions: Both PCS and PainDETECT were found to be poor predictors of postoperative pain, LOS and other immediate postoperative outcomes following TJA.

## 1. Introduction

Total knee arthroplasty (TKA) and total hip arthroplasty (THA) are two surgical procedures with a steadily increasing demand; the number of each of these procedures is growing substantially each year

Surgical techniques and prostheses have improved in recent years, and outcomes after TKA and THA are constantly improving. However, some patients have a lower postoperative improvement in pain, physical function, and quality of life and report dissatisfaction with the outcomes of their TKA or THA [1,2]. The incidence of persistent pain after THA and TKA was reported to be as high as 38% and 53%, respectively [3]. These suboptimal results cannot be entirely explained by medical status variables, such as adverse events and physical comorbidities, but seem to be related to other psychological and social aspects of the patient or the procedure [4].

Over the past few decades, the pain literature has extensively focused on the association between cognitive factors and pain processing. Of the cognitive coping measures, pain catastrophizing (the tendency to focus on and magnify pain sensations and feel helpless in the face of pain) has consistently been demonstrated to be highly predictive of higher levels of pain perception, psychological distress, impaired function, analgesic use and overuse, pain-related disability, and health service use in clinical and nonclinical samples [5] and was identified as a significant and consistent psychological predictor of pain perception [6]. Pain catastrophizing was also found to represent an independent predictor of poor treatment outcomes, including the development of chronic pain after surgery [7].

Another type of pain known to adversely affect quality of life is neuropathic pain [8]. Neuropathic pain is defined as pain that arises as a direct consequence of a lesion or disease affecting the somatosensory system and that does not usually respond to simple analgesics [9]. PainDETECT is a validated questionnaire developed to discriminate between nociceptive and neuropathic pain. Previous studies have shown PainDETECT to be a reliable discriminative tool to identify knee and hip osteoarthritis patients with a neuropathic pain profile [10]. In a recent study, Power et al. demonstrated that a notable proportion of end-stage knee and hip OA patients have pain with potential neuropathic features, with 18.4% and 13.9% of the sample having scores representative of possibly or likely neuropathic pain, respectively [11]. In the same study, higher pain scores, including pain catastrophizing scores (PCS), were independently associated with higher neuropathic pain scores. Both PCS and PainDETECT scores were found to correlate with postoperative pain and functional outcomes in TJA [7,12,13]. However, little data exist concerning PCS and PainDETECT’s ability to predict immediate outcomes following surgery.

The primary objective of this prospective study was to examine the relationship between preoperative pain catastrophizing and neuropathic pain and the immediate outcomes following TJA, including postoperative pain, length of hospital stay (LOS), distance walked during the hospital stay, rate of readmission, discharge destination and early postoperative complications. We hypothesize that higher preoperative PCS and neuropathic pain scores will correlate with poor immediate outcomes as described above.

## 2. Methods

A prospective, observational cohort study of patients undergoing primary total hip (THA) and knee arthroplasty (TKA) was conducted at a single academic institution from December 2015 to July 2016. The study protocol was approved by the respective center’s institutional review board and no funding was provided for this study. Patients were recruited from three participating surgeons’ (CSM, MJB, DC) patient populations after being evaluated at the Joint Replacement Center’s outpatient clinics. Candidates for TJA were screened for eligibility, and their informed consent was obtained. The data were collected using a standardized protocol. Preoperative data were collected within the six weeks before the TJA procedure, and follow-up data were collected during the hospital stay, 30 days and 90 days following surgery. The clinical data following discharge were gathered from the patients’ electronic medical records.

The inclusion criteria required that the arthroplasty patients be older than 18 years, undergoing unilateral primary THA or TKA and able to comply with instructions. All patients undergoing revision surgery or trauma were excluded. We also excluded patients with a diagnosis of depression or active treatment with an antidepressant or anxiolytic, as well as patients with a musculoskeletal diagnosis that could potentially interfere with their interpretation of pain (fibromyalgia, spinal stenosis, significant ipsilateral hip/knee OA). Demographics were recorded preoperatively, and a Charlson comorbidity score was determined [7]. The Pain Catastrophizing Scale (PCS) is a 13-item thought scale designed to identify patients who tend to catastrophize pain and is frequently used in OA populations [11,14].

Patients are asked to rate how often they experience each of the 13 thoughts when they are in pain [4,15]. Thoughts are rated on a 5-point scale of frequency, with 0 being “not at all” and 4 being “all the time”. The PCS yields a total score as well as three subscale scores (rumination, magnification, and helplessness) that make up the multidimensional construct of catastrophizing. In order to simplify the analysis and the interpretation of the results, we considered only the total PCS score in this study. A score of 30 or greater on the PCS is considered a reliable threshold with clinically relevant influence and high internal consistency [16], as it corresponds to the 75th percentile of scores in clinical samples of chronic pain patients. This cutoff value was used to define patients as ‘catastrophizers‘ or ‘non-catastrophizers’.

PainDETECT is a score system developed to discriminate between nociceptive and neuropathic pain and was previously shown to be a reliable discriminative tool to identify knee and hip osteoarthritis patients with a neuropathic pain profile [10]. It uses a combination of a visual analog scale, body diagram, and Likert-type questions to ask about the everyday frequency of symptoms, such as “electric shocks” or “painful light touch.” A total score is calculated, with participants scoring ≥19 defined as “positive neuropathic” [10].

The Western Ontario and McMaster University Osteoarthritis Index (WOMAC) was used to assess clinical health status relevant to TKA outcomes. The WOMAC is an instrument that yields a total score and subscale scores for (1) Pain, (2) Stiffness, and (3) Physical Function [17]. Higher scores on the WOMAC indicate worse pain, stiffness, and functional limitations.

The PCS, PainDETECT and WOMAC scores were obtained during the final preoperative visit, approximately 1–2 weeks prior to their scheduled surgery.

All THA patients had spinal anesthesia and all TKA patients had spinal anesthesia with an adductor nerve block. All TJA patients received preoperative celecoxib (200 mg BID, unless contraindicated), gabapentin and acetaminophen (1000 mg) as is standard care as part of the hospital’s orthopedics-anesthesia joint replacement center. Post-operatively, patients were placed on standing acetaminophen, gabapentin/pregabalin and celecoxib if not contraindicated, along with PRN oxycodone (2.5/5/10 mg) based on their VAS.

All surgeries were performed by three high-volume arthroplasty surgeons at a single center. The surgery was performed under spinal anesthesia without the use of intrathecal narcotics. TKA procedures all used a medial parapatellar approach, using a cruciate-sacrificing total knee in all patients (31 Nexgen; Zimmer, Warsaw, IN, USA; 13 Attune; DePuy, Raynham, MA, USA; 9 Persona; Zimmer, Warsaw, IN, USA). A standard extramedullary guide was used for the proximal tibia cut, whereas intramedullary guides were used to facilitate the femoral cuts. The surgical approach for THA procedures was a posterior approach, which was used in all 40 cases. All THA procedures used a cementless construct (23 DePuy Pinnacle acetabular component and Corail cementless stem (Johnson and Johnson, New Brunswick, NJ, USA); 13 DePuy Pinnacle acetabular component and Summit stem (Johnson and Johnson, New Brunswick, NJ, USA); and 9 Stryker Tritanium acetabular component and Stryker Accolade II cementless femoral stem (Stryker, Mahwah, NJ, USA)).

The two primary outcome measures were the length of hospital stay and discharge destination. Secondary outcome measures included patient-reported postoperative pain at several time points as measured on a 0–10 visual analog scale (VAS), distance walked on POD1, maximal distance walked before discharge, early postoperative complications, and 30 and 90-day readmission rates. The primary pain outcome time point was postoperative VAS at 4 weeks.

### Statistical Analysis

The sample size was chosen to be 100 patients based on the clinical volume of the study and the surgeons’ expectation of a 10% follow-up loss. A post-hoc analysis showed that 90 patients would more than adequately power a comparison between PCS subgroups, assuming a 2:1 ratio of catastrophizers to non-catastrophizers with an alpha of 0.05 to 80% power. Continuous variables were analyzed using a paired Student’s t-test after testing for normality and equal variance. Categorical analysis was conducted with chi-square and Fisher’s exact test where appropriate. A P value of less than or equal to 0.05 was treated as statistically significant.

Multivariable regression analyses were implemented to investigate the possible predictors for the LOS, discharge destination, and early postoperative complications. Variables in the regression analysis included BMI, gender, age, type of surgery (THA/TKA), Charlson comorbidity, preoperative WOMAC, preoperative PCS and PainDETECT scores, postoperative VAS scores and distances walked on postoperative day (POD) 1, use of chronic pain medications and maximal distance walked before discharge. Potential predictive factors that reached a level of significance were *p* < 0.10 after univariate analysis was included in a multivariate regression model. All analyses were performed using SPSS (SPSS 24.0, IBM Inc., Somers, NY, USA). A complete case analysis was performed to account for missing data.

## 3. Results

During the study period, 100 patients agreed to participate in the study and completed the relevant scores following their initial evaluation. Complete clinical data were available for 93 patients who underwent elective TJAs (40 THAs and 53 TKAs). The sample’s mean age was 67 years (SD: 10.4) and 53% were men. The prevalence of pain catastrophizing (PCS ≥ 30) and neuropathic pain (PainDETECT ≥ 19) was 45% and 20.4%, respectively. The mean PCS did not differ between THA and TKA patients and was 24.3 ± 16 and 28.2 ± 17.6, respectively (*p* = 0.27). Likewise, the mean PainDETECT score did not differ significantly between THA and TKA patients and was 10.9 ± 7.7 and 11.8 ± 8.6, respectively (*p* = 0.6).

Pain catastrophizers had similar demographics and clinical characteristics to non-catastrophizers (Table 1), except for the preoperative WOMAC which was significantly higher in pain catastrophizers (77.28 ± 13.46 vs. 58.51 ± 16.68, *p* < 0.001), and the preoperative (day 0) VAS was significantly higher in pain catastrophizers (6.29 ± 3 vs. 4.5 ± 2.6, mean difference 1.73, 95% CI 0.57–2.9, *p* = 0.004). However, the VAS at POD1, POD2 and 4 weeks did not significantly differ between groups. Similarly, the distance walked on POD1, maximal distance walked during the hospital stay, the discharge destination, early postoperative complications and readmissions did not differ significantly between the groups. The LOS was longer for pain catastrophizers (78.8 vs. 68.2 h, mean difference 10.6 h, 95% CI −6.8–28.1), although this difference was not statistically significant (*p* = 0.23).

After analyzing the results for neuropathic pain using the threshold value of 19 on the PainDETECT scale, we found statistically significant differences in the preoperative WOMAC and preoperative (day 0) VAS between the two groups (Table 2). The preoperative WOMAC was found to be significantly higher in the neuropathic group 83.2 ± 9.1 vs. 62.8 ± 17.2 (*p* < 0.001), and the preoperative VAS was 7.58 ± 2.2 compared to 4.76 ± 2.7 (*p* < 0.001), respectively.

The postoperative VAS at 4 weeks following the index procedure was significantly higher in patients with neuropathic pain compared to patients without such pain (9.3 ± 0.88 vs. 7.68 ± 2.4, respectively, *p* = 0.006). The LOS, maximal distance walked on POD1, early postoperative complications and readmission rates, and mean VAS on POD1 and POD2 did not vary significantly between the two groups. The maximal distance walked during the hospital stay was less in patients with neuropathic pain (120 ± 83.3 vs. 168.5 ± 101.5, mean difference −48.5, 95%CI −98.6–1.6), although this was not significant statistically (*p* = 0.058). The existence of neuropathic pain did affect the discharge destination, with a significantly higher proportion of patients necessitating referral to rehabilitation than patients less likely to have neuropathic pain (47.3% vs. 17.5%, *p* = 0.02).

A linear regression model (Supplementary Table S1) was used to look for potential predictors of the LOS, controlling for gender, BMI, age, type of surgery, PCS, WOMAC, PainDETECT, Charlson Comorbidity, mean VAS postoperative, LOS, distance walked POD1 and discharge destination. None of the factors were found to be of statistical significance.

Another model (Supplementary Table S2) looking at early postoperative complications showed that the only factor affecting their occurrence was the use of chronic pain medication (*p* = 0.039, Odds ratio 48.95, 95% CI (1.22 to 1961.1), bivariate regression, model fit *p* = 0.011).

An ordinal regression analysis (Supplementary Table S3) was used to find possible predictors for discharge destination (rehabilitation vs. home). The PCS, PainDETECT, WOMAC, age, BMI, chronic pain medications, and postoperative complications were all non-significant. The only finding remaining statistically significant was the distance walked on POD1 (*p* < 0.001, Odds ratio −0.082 95% CI (−0.125 to −0.039)). PCS and PainDETECT were positively correlated with an rs = 0.501 (*p* = 0.001) on Spearman’s test. WOMAC was positively correlated more strongly with PCS (rs = 0.512 *p* = 0.01) than with PainDETECT (rs = 0.329 *p* = 0.038) on Spearman’s test.

## 4. Discussion

Despite the advances in surgical techniques, regional anesthesia, improved postoperative pain management with multimodal and preemptive analgesia, along with rapid rehabilitation protocols, there is still a significant proportion of dissatisfied patients despite undergoing a well-performed TJA procedure. Most studies dealing with patient dissatisfaction after TJA report either combined outcomes for patients undergoing both THA and TKA or report on outcomes specific to TKA [1,2,18]. In these studies, up to 15% of patients report some degree of dissatisfaction after surgery. Dissatisfaction is evidently multifactorial, but a contributing factor is certainly pain [3].

The ability to pre-operatively identify patients who are less likely to improve following their surgery may be critical for optimal management, patient selection and well-organized service delivery. These essential data could act as an adjunct to clinical and radiographic data for surgeons and healthcare providers who refer patients for surgery. In addition, these data could help surgeons to counsel patients regarding what they may expect in terms of pain and function following surgery and could help the patient to set realistic expectations. Although the literature for psychological interventions (cognitive behavioral therapy, psycho-education, motivational interviewing, relaxation therapy and guided imagery) in conjunction with TKA and THA is still in its infancy [19], identifying patients who may benefit from such interventions can perhaps lead to better utilization of these treatment options in the future, along with improved postoperative pain outcomes. 

Our findings suggest that a notable proportion of end-stage knee and hip osteoarthritis patients who are considered potential candidates for arthroplasty procedures have pain with potential neuropathic features or catastrophizing pain. This finding is in line with a recent study by Power et al. who found an incidence of neuropathic pain reaching 19.4% in women with end-stage OA [11]. They also found a mean PCS of 25 and a mean PainDETECT of 10. 4, which was very similar in our cohort (mean PCS 26.5 ± 17 and mean PainDETECT 11.4 ± 8.2). Interestingly, our results did not reflect significant differences in pain scores depending on the type of surgery or gender.

The LOS In the hospital for TJA has been declining in recent decades [20]. Preoperative prediction of the LOS following TJA is essential for operational efficiency, management of the patient’s expectations, and reduction in healthcare costs. Several factors were found to be associated with a prolonged LOS, among which are an increased number of comorbidities, lack of adequate caregiver assistance at home, and bilateral surgery [21]. Our findings do not suggest that any of the scores used could be considered a predictor of a lengthier hospital stay. The distance walked on POD1, an important physical therapy milestone, remained the only significant predictor of discharge destination in a multivariate analysis. This finding is in line with a previous study by Sharareh et al. [22] that showed that patients who were ultimately discharged to a skilled nursing facility walked less than half the distance, on average, than patients who were discharged to home.

PainDETECT differentiates between nociceptive and neuropathic pain and was previously found to be a reliable discriminative tool to identify knee and hip osteoarthritis patients with a neuropathic pain profile [10]. An important finding of the present study is that patients showing a likely neuropathic pain profile (PainDETECT ≥ 19) are significantly less likely to be discharged home following their surgery, with nearly 48% requiring transfer to an inpatient rehabilitation facility. Identifying the independent drivers of a non-home discharge destination is critical to understanding the decision process of patients and their surgeons before surgery to reduce rates of non-home discharge.

However, neither PainDETECT nor PCS remained significant predictors of discharge destination in a multivariate analysis.

We acknowledge several limitations of the present study. First, we did not include evaluations of baseline mental health, which has been previously shown to affect various aspects of functional recovery after TJA [23]. Second, PCS and PainDetect were obtained only once, although it was previously shown that pain-related catastrophizing is a dynamic construct that is related to pain intensity rather than a static trait [5]. Third, despite taking into consideration the use of preoperative chronic pain medications, we did not control for postoperative opioid intake. Fourth, we are not able to correct for multiple comparison data, which may limit the strength of our multivariate regression model. Lastly, we did not incorporate other available tools for predicting the risk of non-home discharge in our analysis. Furthermore, this study was conducted at a single center with a limited number of patients.

## 5. Conclusions

In this prospective cohort study, we sought to determine whether PainDETECT and PCS could be used as predictors of postoperative pain and immediate outcomes following TJA. PCS was found to be of limited usefulness in predicting the immediate postoperative pain and outcomes following TJA. However, our findings suggest that a subset of patients with a substantial preoperative neuropathic pain component, detected with a PainDETECT equivalent of 19 or greater have higher pain levels at 4 weeks and are significantly more likely to be discharged to a skilled nursing facility.

## Figures and Tables

**Table 1:** Pre- and postoperative characteristics of cohort subjects, pain catastrophizers and non-catastrophizers.

Type	PCS < 30	PCS ≥ 30	*p* Value
	(*n* = 51)	(*n* = 42)	
Age (years, mean ± SD)	68.6 ± 9.9	66.2 ± 11	0.27
Gender (% female)			
BMI (mean ± SD)	30.5 ± 6.8	31.2 ± 7	0.62
Charlson comorbidity score	2.92	3.15	0.43
Chronic pain medications	4 (7.8%)	6 (14.6%)	0.33
Pre-operative WOMAC	58.51 ± 16.68	77.28 ± 13.46	<0.001
Indication for surgery			0.33
Osteoarthritis	49.00	40.00	
AVN	2.00	0.00	
RA	1.00	2.00	
Type of surgery			0.21
TKA	26.00	27.00	
THA	25.00	15.00	
Preoperative ambulatory status			0.74
Independent	41.00	36.00	
Cane	9.00	5.00	
Walker	1.00	1.00	
VAS			
Day 0	4.55 ± 2.6	6.29 ± 3	0.004
Day 1	5.2 ± 2.9	5.78 ± 2.7	0.32
Day 2	3.92 ± 2.6	4.58 ± 3	0.28
VAS (4 weeks)	7.78 ± 2.2	8.29 ± 2.4	0.3
Distance walked POD1	78.7 ± 68.4	78.8 ± 74.5	0.99
Maximal distance walked before Discharge	151.9 ± 68.2	166.6 ± 96	0.48
LOS (hours)	68.2	78.8	0.23
Discharge destination			0.46
Home	38 (74.5%)	29 (69%)	
Acute rehabilitation	1 (1.9%)	3 (7.1%)	
Rehabilitation	12 (23.5%)	10 (23.8%)	
Early post-operative complications	6 (11.7%)	3 (7.14%)	0.5
30-days readmission rate	1 (1.9%)	0 (0%)	0.36
90-days readmission rate	1 (1.9%)	0 (0%)	0.36

**Table 2:** Pre- and postoperative characteristics of patients with and without neuropathic pain.

Type	PainDETECT < 19	PainDETECT ≥ 19	*p* Value
	(*n* = 74)	(*n* = 19)	
Age (years, mean ± SD)	68 ± 10	66 ± 12	0.47
Gender (% female)	50.00	52.60	0.83
BMI (mean ± SD)	31.1 ± 7.1	29.7 ± 5.7	0.4
Charlson comorbidity score	3 ± 1.3	3 ± 1.5	0.91
Chronic pain medications	6 (8.1%)	4 (21%)	0.2
Pre-operative WOMAC	62.8 ± 17.2	83.2 ± 9.1	<0.001
Indication for surgery			0.51
Osteoarthritis	71 (96%)	17 (89.5%)	
AVN	1 (1.3%)	1 (5.2%)	
RA	2 (2.6%)	1 (5.2%)	
Type of surgery			0.61
TKA	41 (55.4%)	12 (63.2%)	
THA	33 (44.6%)	7 (36.8%)	
Preoperative ambulatory status			0.29
Independent	59.00	18.00	
Cane	13.00	1.00	
Walker	2.00	0.00	
VAS			
Day 0	4.76 ± 2.7	7.58 ± 2.2	<0.001
Day 1	5.3 ± 2.9	6 ± 2.5	0.3
Day 2	4 ± 2.8	4.94 ± 2.77	0.22
VAS (4 weeks)	7.68 ± 2.4	9.3 ± 0.88	0.006
Distance walked POD1	82.3 ± 70.6	65 ± 72	0.34
Maximal distance walked before Discharge	168.5 ± 101.5	120 ± 83.3	0.058
LOS (hours)	73.6 ± 45.4	70.8 ± 28.7	0.8
Discharge destination			0.02
Home	57 (77%)	10 (52.6%)	
Acute rehabilitation	4 (5.4%)	0 (0%)	
Rehabilitation	13 (17.5%)	9 (47.36%)	
Early post-operative complications	6 (12.1%)	0 (0%)	0.11
30-days readmission rate	1 (1.35%)	0 (0%)	0.61
90-days readmission rate	1 (1.35%)	0 (0%)	0.61

BMI: body mass index; SD: standard deviation; WOMAC: Western Ontario and McMaster Universities Osteoarthritis Index; AVN: avascular necrosis; RA: rheumatoid arthritis; POD: postoperative day; VAS: visual analog scale; LOS: length of stay.

## Data Availability

The data presented in this study are available on request from the corresponding author. The data are not publicly available due to privacy and ethical reasons.

## Supplementary Materials

The following supporting information can be downloaded at: https://www.mdpi.com/article/10.3390/jpm13020216/s1, Table S1: Linear regression model, predictors of LOS; Table S2: Bivariate regression model, post-operative complications; Table S3: Ordinal regression analysis, predictors of discharge destination.
